# Evaluation of an adaptive, rule-based dosing algorithm to maintain therapeutic anticoagulation during atrial fibrillation ablation

**DOI:** 10.1016/j.cvdhj.2023.11.001

**Published:** 2023-11-14

**Authors:** Matthew M. Kalscheur, Matthew R. Martini, Marcus Mahnke, Fauzia Osman, Daniel S. Modaff, Blake E. Fleeman, Ryan T. Kipp, Jennifer M. Wright, Joshua E. Medow

**Affiliations:** ∗ Division of Cardiovascular Medicine, Department of Medicine, University of Wisconsin School of Medicine and Public Health, Madison, Wisconsin; † Integrated Vital Medical Dynamics, LLC, Madison, Wisconsin; ‡ Department of Medicine, University of Wisconsin School of Medicine and Public Health, Madison, Wisconsin; § Departments of Neurosurgery, Neurology, and Biomedical Engineering, Medical College of Wisconsin, Milwaukee, Wisconsin

**Keywords:** Atrial fibrillation, Catheter ablation, Clinical decision support system, Anticoagulation, Implementation, Quality improvement

## Abstract

**Background:**

Cerebral thromboembolism during atrial fibrillation (AF) ablation is an infrequent (0.17%) complication in part owing to strict adherence to intraprocedural anticoagulation. Failure to maintain therapeutic anticoagulation can lead to an increase in events, including silent cerebral ischemia.

**Objective:**

To evaluate a computerized, clinical decision support system (CDSS) to dose intraprocedural anticoagulation and determine if it leads to improved intraprocedural anticoagulation outcomes during AF ablation.

**Methods:**

The Digital Intern dosing algorithm is an adaptive, rule-based CDSS for heparin dosing. The initial dose is calculated from the patient’s weight, baseline activated clotting time (ACT), and outpatient anticoagulant. Subsequent recommendations adapt based on individual patient ACT changes. Outcomes from 50 cases prior to algorithm introduction were compared to 139 cases using the algorithm.

**Results:**

Procedures using the dosing algorithm reached goal ACT (over 300 seconds) faster (17.6 ± 11.1 minutes vs 33.3 ± 23.6 minutes pre-algorithm, *P* < .001). ACTs fell below goal while in the LA (odds ratio 0.20 [0.10–0.39], *P* < .001) and rose above 400 seconds less frequently (odds ratio 0.21 [0.07–0.59], *P* = .003). System Usability Scale scores were excellent (96 ± 5, n = 7, score >80.3 excellent). Preprocedure anticoagulant, weight, baseline ACT, age, sex, and renal function were potential predictors of heparin dose to achieve ACT >300 seconds and final infusion rate.

**Conclusion:**

A heparin dosing CDSS based on rules and adaptation to individual patient response improved maintenance of therapeutic ACT during AF ablation and was rated highly by nurses for usability.

## Graphical abstract

**Figure:**
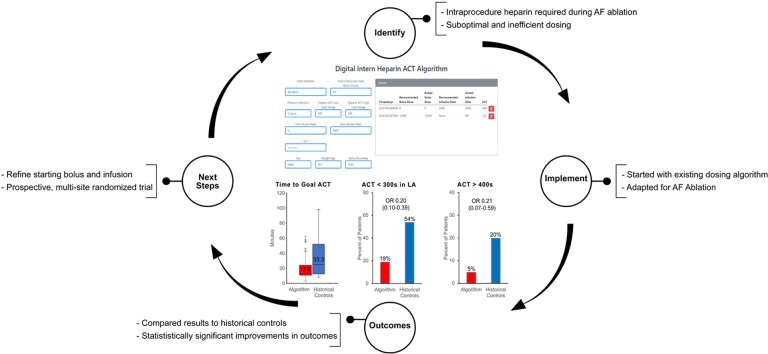

## Introduction

With the growing burden of atrial fibrillation (AF), AF ablation volume is increasing.^1^ Catheter ablation for AF has emerged as the standard approach for invasive management of AF and is the most common catheter-directed ablation procedure performed today.^2^ However, catheter ablation carries a risk for serious complications, including cerebrovascular events. Minimizing the risk of these events through strict intraprocedural anticoagulation is essential.^3^

Catheter ablation requires transseptal puncture and placement of 1 or 2 sheaths in the left atrium that remain across the interatrial septum for the duration of the ablation procedure. This procedure exposes patients to periprocedural bleeding and cerebral thromboembolic events, necessitating optimal anticoagulation management before, during, and after the procedure.^4^, ^5^, ^6^, ^7^ To reduce these risks, data support continuing uninterrupted anticoagulation with warfarin or non–vitamin K antagonist oral anticoagulants (NOACs), administration of intravenous unfractionated heparin prior to transseptal puncture, and maintenance of intraprocedural activated clotting times (ACT) >300 seconds.^8^, ^9^, ^10^, ^11^

Recent reports have demonstrated unpredictable and highly variable initial levels of anticoagulation using NOACs.^2^^,^^12^^,^^13^ At the University of Wisconsin, an academic center in Madison, Wisconsin, with 5 electrophysiologists performing catheter ablation during the study period, heparin dosing was inconsistent between providers and required frequent nurse-physician interactions for dosing decisions. Others have reported on similar knowledge gaps in catheter ablation for AF: (1) difficulty achieving therapeutic ACT, especially with NOACs; (2) practice variation among individual providers; and (3) lack of protocols to deliver intraprocedural anticoagulation.^14^, ^15^, ^16^, ^17^ The protocols described in these prior reports did not include a computer-based clinical decision support system (CDSS).

A well-designed computer-based CDSS can improve patient care.^18^ At the University of Wisconsin, a computer-based CDSS has significantly improved patient blood management.^19^ The electrophysiology team at the University of Wisconsin hypothesized that a similar system could improve intraprocedural anticoagulation. Here, we report the results of a retrospective evaluation of that computerized, adaptive, rule-based heparin dosing algorithm to determine the impact on time to goal ACT and the number of procedures with out-of-range ACTs during AF catheter ablation compared to historical controls.

## Methods

The Digital Intern® (Integrated Vital Medical Dynamics [iVMD], Madison, WI) is a collection of computer algorithms developed to support clinical care. A Digital Intern module contributed to guideline-based standardization of red blood cell transfusion at the University of Wisconsin.^19^ The Digital Intern procedure–based heparin dosing calculator supports heparin dosing for patients during interventional cardiology and neuroendovascular procedures. This algorithm was modified and evaluated to support heparin dosing during electrophysiologic procedures from January 2021 through October 2021 as part of a quality improvement effort. Here, we retrospectively evaluated the impact of this modified module, ie, “dosing algorithm,” on maintenance of guideline-based therapeutic ACT during AF ablation. This retrospective review received approval from the University of Wisconsin School of Medicine and Public Health Institutional Review Board (IRB 2022-0312).

### Dosing algorithm development

**Figure 1:**
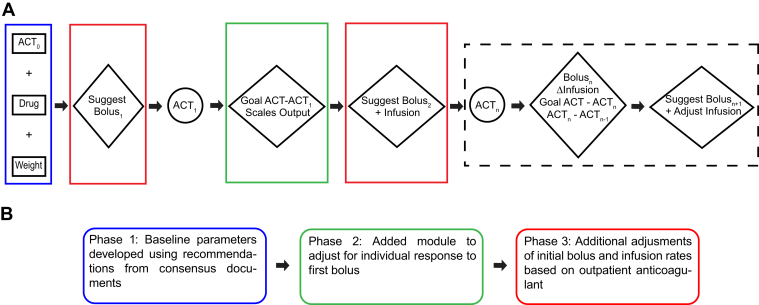
Description and development of the heparin dosing algorithm. **A:** The Digital Intern (Integrated Vital Medical Dynamics, Madison, WI) procedure–based heparin dosing calculator, ie, “dosing algorithm,” is an adaptive, rule-based system that mathematically generates recommendations based on the distance from current activated clotting time (ACT) to the central target. To initiate the algorithm, initial bolus and infusion recommendations are required. Bolus_1_ is determined based on patient weight, type of anticoagulant, and distance from goal ACT to ACT_0_. The individual patient response to Bolus_1_ scales the coefficients that generate all future bolus and infusion recommendations. From ACT_2_ onward, the previous bolus, change in infusion rate, (Goal ACT - ACT_n_), and (ACT_n_−ACT_n__−__1_) continue to refine the coefficients until steady state is achieved. Text in black boxes represent initial user inputs, those in circles are subsequent user inputs, and text in rhombi represent actions of the dosing algorithm. **B:** The algorithm was developed in 3 phases. Baseline performance was established using recommendations from consensus documents and institutional experience to initiate the algorithm (*blue boxes*). Next, a module was added to account for significant variation in response to the initial bolus (*green boxes*). Finally, adjustments were made in the initial bolus amount and infusion rate (*red boxes*).

The dosing algorithm is a rule-based, deterministic system that mathematically generates recommendations based on the distance of the current ACT to the goal ACT (Figure 1). These rules are represented in a mathematical function and use individual patient response to previous recommendations to refine future recommendations. An initial bolus is calculated based on patient weight, baseline ACT, oral anticoagulant, and goal ACT. At specified times, staff obtain additional ACTs. An infusion rate is calculated after obtaining the first postbolus ACT. Each bolus dose and infusion rate change paired with subsequent ACT measurements continues to refine the coefficients in the function that generates each recommendation to meet the needs of a specific patient. Those coefficients are adjusted until steady state is achieved. The specific mathematical function and coefficients that produce the adaptive adjustments are proprietary and not specified here. A Q-submission (a request for feedback and meetings prior to medical device submission) was prepared by iVMD and reviewed by the US Food & Drug Administration (FDA). As the product provides recommendations supported by medical literature and reviewed by medical personnel, the FDA ruled that no regulation of this product was required.

Modification of the product for the electrophysiology lab occurred in 3 phases (Figure 1). Based on prior institutional experience and published data, initial heparin bolus and infusion rates were selected (120 units/kg in patients anticoagulated on an uninterrupted direct oral anticoagulant, 75 units/kg in patients anticoagulated with warfarin, and 100 units/kg in patients on no anticoagulation).^2^^,^^11^^,^^20^ To initiate the protocol, an ACT was drawn (ACT+ assay; Hemochron Signature Elite ACT machine; Werfen, Bedford, MA). The standard bolus amount was adjusted downward proportionate to the proximity of the baseline ACT to the goal ACT. In all patients, the initial infusion was 18 units/kg for NOACs and 15 units/kg for warfarin.

The second phase added an adjustment for variable responses to the initial heparin bolus. In the first month of development, approximately 50% of patients reached an ACT >300 seconds after the initial bolus (Supplemental Table 1). This observation led to the addition of an adaptive module, which modified subsequent boluses and the heparin infusion rate, scaling up subsequent recommendations if the first ACT post bolus was less than goal.

In the third phase, infusion rates were increased based on the final rates observed (Supplemental Table 2), and the bolus amount for dabigatran was decreased. Dabigatran data were limited, but the postbolus ACT was consistently >400 seconds using a bolus amount of 120 units/kg. Final baseline bolus and infusion rates were as follows: apixaban, bolus = 120 units/kg, infusion 27 units/kg; rivaroxaban, bolus = 120 units/kg, infusion 25 units/kg; dabigatran, bolus 90 units/kg, infusion 18 units/kg; warfarin, bolus 75 units/kg, infusion 24 units/kg; no anticoagulation, bolus = 120 units/kg, infusion 24 units/kg.

### Catheter ablation and intraprocedural anticoagulation management

Catheter ablation was performed using standard techniques.^2^ Patients presented in the fasted state and were placed under general anesthesia. Anticoagulation was uninterrupted. Ablations were completed with 1 or 2 transseptal sheaths with heparin infusions flowing through those sheaths. Bolus heparin was given by anesthesia (1000 units/mL concentration), first administered following venous sheath insertion, and the heparin infusion was initiated after the first post-bolus ACT, prior to transseptal puncture.

**Figure 2:**
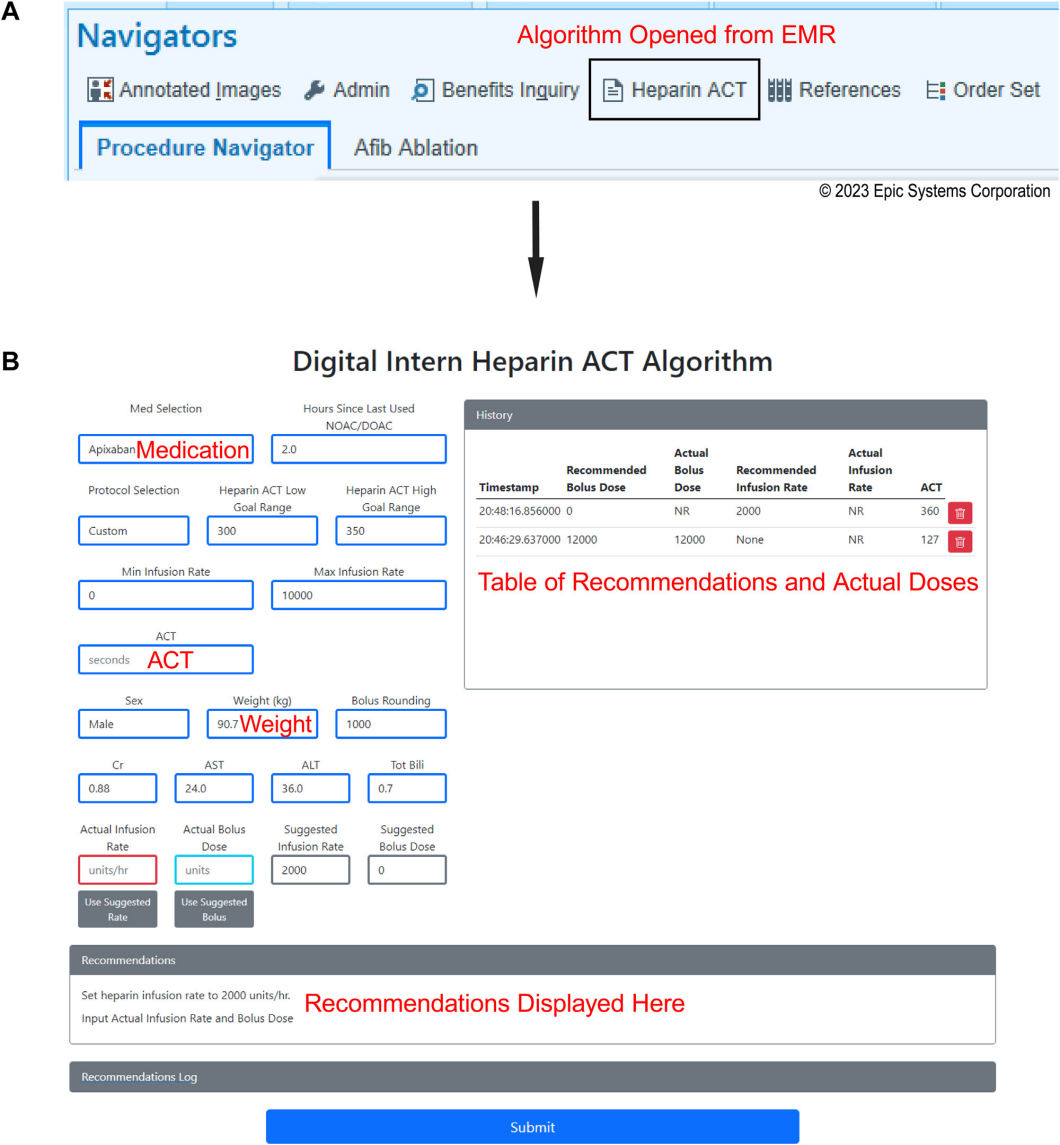
Heparin dosing algorithm initiation and use. **A:** The heparin dosing algorithm was opened by staff directly from the electronic medical record. **B.** Staff then input required data. Patient characteristics input by staff and used in the algorithm calculations included medication used for thromboembolic prophylaxis, weight, and baseline ACT (highlighted in figure). Additional characteristics input included time since last dose of anticoagulant, sex, creatinine, AST, ALT, and total bilirubin. An initial bolus dose was then suggested. Staff entered subsequent activated clotting time results and received recommendations on bolus and infusion rates. Actual doses delivered were tabulated.

Nurses interacted with the dosing algorithm through a web-based application programming interface accessed through the electronic medical record (EMR) (Figure 2). Inputs included anticoagulant, goal ACT (300–350 s for all left atrial ablations), hours since last dose of medication, sex, weight, creatinine, AST, ALT, total bilirubin, and a baseline ACT. No patient identifiers are entered into the interface. The dosing algorithm calculations are performed on an Amazon Web Services instance managed by iVMD. As shown in Figure 2, the dosing algorithm recommendations are then returned to the staff through the same interface. Staff input the bolus and infusion rates chosen and resulting ACTs. Nursing staff drew ACTs every 10–15 minutes initially based on suggestions from the dosing algorithm. If 3 consecutive ACTs were stable at goal ACT, duration was extended to 30 minutes. Importantly, nurses provided oversight of the ACTs. The ACT+ coefficient of variation is reported at ≤10%.^21^ If any ACT seemed inaccurate, it was redrawn for confirmation.

### Study design

Outcomes related to intraprocedural heparin dosing in 50 consecutive historical controls (September 2020 through December 2020) were compared to outcomes in 139 cases performed using the dosing algorithm (October 2021 through February 2022). Primary outcomes included time to ACT >300 seconds, number of patients with any ACT >400 seconds, and number of patients with any ACT <300 seconds while operating in the left atrium. Nurses using the algorithm completed the 100-point System Usability Scale (SUS) assessment and provided qualitative assessments. The SUS provides a widely used, “quick and dirty” tool for measuring reliability of hardware, software, mobile devices, websites, and applications.^22^ For dosing algorithm cases, predictors of the total heparin bolus amount required to achieve an ACT >300 seconds and the final heparin infusion rate were modeled.

### Data collection

Data entered into the dosing algorithm and recommendations were stored in an Amazon Web Services database accessible by iVMD and available to the University of Wisconsin team (no patient identifiers present in these data). All ACT readings, bolus amounts, and infusions were also stored in the University of Wisconsin EMR as part of the clinical record. Under IRB approval, EMR data were accessed for patients included in the study and datasets were developed through chart review by the University of Wisconsin team (M.M.). Deidentified datasets developed through chart review were used for analysis (F.O.) and are available for review upon request. Only cardiology staff from the University of Wisconsin had access to both datasets and the outcomes.

### Statistical analysis

**Figure 3:**
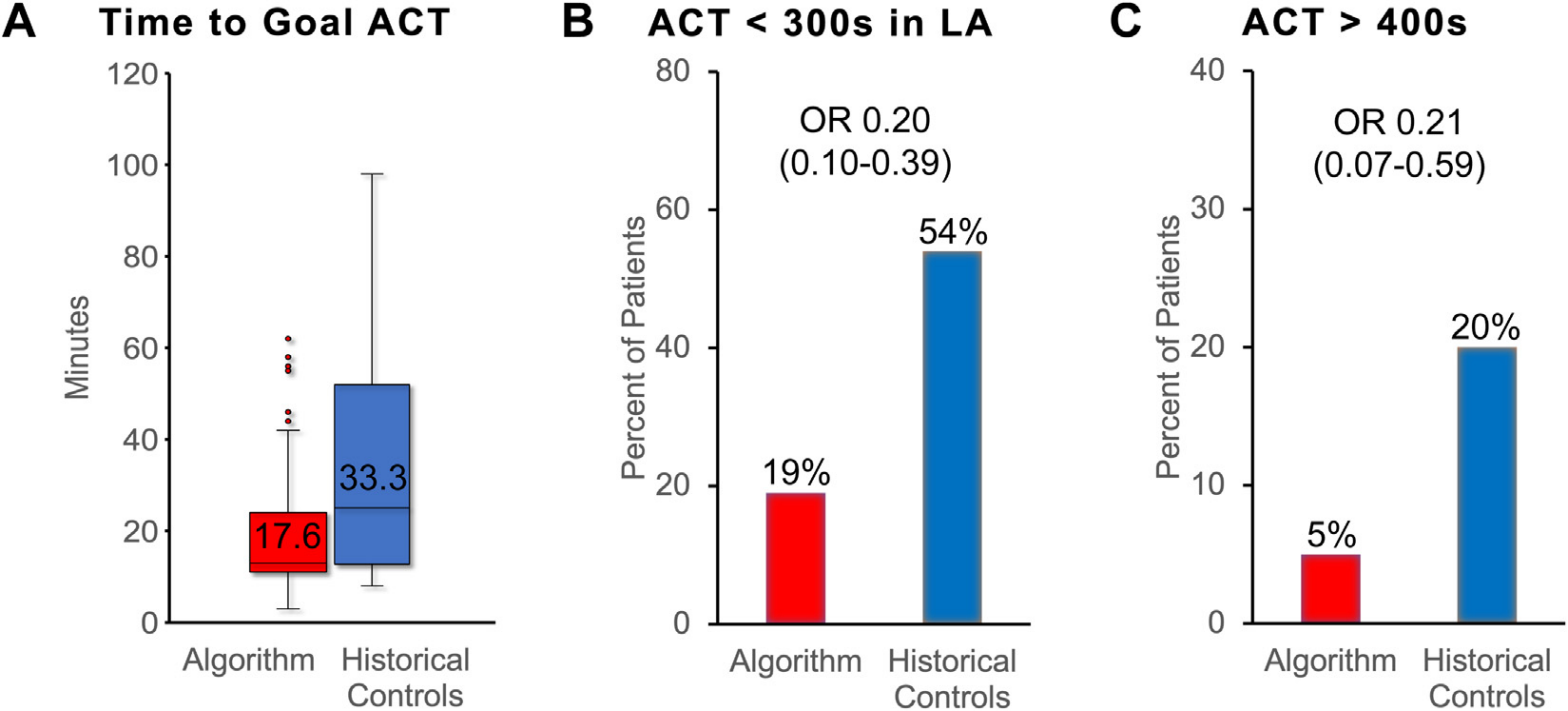
Intraprocedural anticoagulation outcomes improved with the dosing algorithm. **A:** The time to reach goal activated clotting time (ACT) dropped from 33.3 ± 23.6 minutes to 17.6 ± 11.1 minutes, beta coefficient for linear regression -15.8, confidence interval (CI): -20.8 to -10.8, *P* < .001. In this box plot, the box covers the interquartile range while the whiskers are restricted to 1.5 times the interquartile range; outliers are defined as greater than 1.5 times the interquartile range. **B:** The percentage of patients experiencing ACT <300 seconds while operating in the left atrium dropped from 54% (27/50 historical controls) to 19% (26/139 dosing algorithm), odds ratio (OR) 0.20, CI: 0.10–0.39, *P* < .001. **C:** The percentage of patients with any ACT >400 seconds decreased from 20% (10/50 historical controls) to 5% (7/139 dosing algorithm), OR 0.21, CI: 0.07–0.59, *P* = .003.

We used descriptive analysis to summarize the study data, including frequencies and counts for categorical data and mean and standard deviation for continuous demographics and outcomes. Categorical variables were compared using χ^2^ tests and Fisher exact tests. We checked for skewness and normality in continuous variables using histogram plots and Bartlett’s tests. We used a Student *t* test for all numerical comparisons, as all data were found to be normally distributed. The outcomes shown in Figure 3 and Supplemental Table 3 were analyzed using linear and logistic regression models to obtain coefficients and odds ratios.

To find predictors of heparin bolus amount to achieve ACT >300 seconds and final heparin infusion rate, we used a generalized linear model to report beta coefficients and 95% confidence intervals. We first conducted a univariate analysis to determine univariate significance and then we included significant predictors from the univariate analysis into a multivariate analysis, adjusting for each factor. Candidate variables included in the univariate analysis were selected based on factors that may influence NOAC dosing (age, weight, renal function, and liver function) and other commonly available variables that may be relevant (baseline ACT, platelets, sex, and body mass index [BMI]). We assessed for multicollinearity between the independent factors using a variance inflation factor. All variables that had a variance inflation factor of 10 or greater were excluded from the adjusted model. Since BMI and weight were directly related, we included only the weight variable to avoid multicollinear estimates. All *P* values that were less than or equal to .05 were considered significant. We conducted our analysis using SAS 9.4 (SAS Institute Inc 2013. SAS/ACCESS® 9.4 Interface to ADABAS: Reference. Cary, NC: SAS Institute Inc).

## Results

### Clinical characteristics

Table 1 includes the characteristics of the 50 historical controls and 139 patients with intraprocedural heparin dosing performed using the dosing. All patients were on uninterrupted thromboembolic prophylaxis at the time of the procedure. There were no significant differences between the groups. The total heparin bolus amount (mean) used per patient was also very similar between historical controls and dosing algorithm cases.

**Table 1:** Characteristics of the study sample

Variable	Historical control (N = 50)	Dosing algorithm (N = 139)	*P* value
Anticoagulation, n (%)			
Warfarin	5 (10.0)	16 (11.5)	.38
Apixaban	34 (68.0)	106 (76.3)	
Rivaroxaban	9 (18.0)	15 (10.8)	
Dabigatran	2 (4.0)	2 (1.4)	
INR (if on warfarin), mean (SD)	2.3 (0.29)	2.4 (0.42)	.12
Age, mean (SD)	62.1 (10.9)	64.4 (9.8)	.17
Sex, n (%)			
Male	28 (56.0)	87 (62.6)	.41
Female	22 (44.0)	52 (37.4)	
Weight (kg), mean (SD)	94.5 (20.0)	98.6 (24.9)	.30
BMI, mean (SD)	31.2 (7.0)	31.8 (6.9)	.60
Creatinine, mean (SD)	0.98 (0.24)	1.02 (0.32)	.42
eGFR, mean (SD)	75.6 (17.2)	74.9 (20.4)	.83
AST, mean (SD)	26.5 (15.2)	25.7 (11.6)	.70
ALT, mean (SD)	30.8 (23.7)	26.8 (16.8)	.20
Tbili, mean (SD)	0.71 (0.37)	0.74 (0.35)	.61
Platelets, mean (SD)	212.7 (35.3)	220.9 (63.6)	.39
Total bolus doses of heparin, mean (SD)	12,430.0 (4844.5)	12,417.3 (4227.1)	.99

ALT = alanine transaminase; AST = aspartate aminotransferase; BMI = body mass index; eGFR = estimated glomerular filtration rate; INR = international normalized ratio; SD = standard deviation; Tbili = total bilirubin.

### Dosing algorithm compared to historical controls

Results comparing dosing algorithm performance to historical controls are shown in Figure 3 and Supplemental Table 3. Using the dosing algorithm, the time to reach goal ACT dropped from 33.3 ± 23.6 minutes to 17.6 ± 11.1 minutes, a statistically significantly change (beta coefficient for linear regression -15.8, confidence interval [CI]: -20.8 to -10.8, *P* < .001). There were also statistically significant reductions in patients experiencing ACT <300 seconds while operating in the left atrium (odds ratio [OR] 0.20, CI: 010–0.39, *P* < .001) or ACT >400 seconds at any point during the procedure (OR 0.21, CI: 0.07–0.59, *P* = .003). In historical controls, 54% of patients (27/50) experienced an ACT <300 seconds while operating in the left atrium compared to 19% (26/139) for the dosing algorithm and 20% (10/50) experienced an ACT >400 seconds compared to 5% (7/139) for the dosing algorithm. When the ACT did drop below 300 seconds while operating in the left atrium, time spent with ACT below goal using the dosing algorithm did decrease, but this was not statistically significant. A high percentage of the dosing recommendations were followed without modification: 98.8% of bolus recommendations and 96.2% of infusion recommendations.

### Qualitative outcomes

Nurses using the algorithm completed the SUS. This 10-item questionnaire is an industry standard for classifying the ease of use of websites and applications. A SUS score above 68 is considered average and a score >80.3 is considered excellent; the dosing algorithm scored 96 (standard deviation 5, n = 7). Nurses and electrophysiologists also provided quotes regarding the dosing algorithm. Positive comments from electrophysiologists included “Fewer distractions during difficult cases, less delay to therapeutic ACT” and “More reliably achieving ACTs, less mental burden on operator.” However, there was some caution expressed as well: “The team just needs to be vocal when an ACT does not increase as expected post heparin so that troubleshooting can occur.” The nurses using the dosing algorithm reported “More autonomy in my practice. Less interruptions for the physician” and “Love the nurse autonomy and consistency.”

### Predictors of heparin doses

For the 139 dosing algorithm cases, regression modeling was performed to determine predictors of the total heparin bolus amount to achieve ACT >300 seconds and the final heparin infusion rate. Using a generalized linear model, warfarin for thromboembolic prophylaxis, age, sex, weight, BMI, estimated glomerular filtration rate (eGFR), and baseline ACT were univariate predictors of total bolus amount to achieve ACT >300 seconds (Table 2). Being on warfarin, weight, and baseline ACT remained statistically significant after multivariate analysis, with eGFR nearly reaching statistical significance. Statistically significant univariate predictors of final infusion rate included being on warfarin (compared to being on apixaban), age, sex, weight, BMI, and eGFR (Table 3). Being on warfarin, sex, and weight remained statistically significant after a multivariate analysis.

**Table 2:** Predictors of heparin bolus amount to achieve activated clotting time >300 seconds

Variable	Univariate			Multivariate		
	β	95% CI	*P* value	β	95% CI	*P* value
Anticoagulation						
Apixaban	Ref	Ref	Ref	Ref	Ref	Ref
Warfarin	-4475.2	-6598.6, -2351.9	<.001∗	-2693.7	-4197.1, -1190.4	.001∗
Rivaroxaban	-437.7	-2621.7, 1746.3	.69	1210.8	-484.6, 2906.3	.16
Dabigatran	-4037.7	-9688.4, 1612.9	.16	-1387.3	-5166.9, 2392.2	.47
INR (if on warfarin)	-5616.2	-9723.8, -1508.7				
Time since last AC dose (h)	2.3	-1.1, 5.6	.19			
Age	-125.7	-195.3, -56.2	<.001∗	-12.6	-68.4, 43.3	.66
Sex = male	2848.1	1458.6, 4237.7	<.001∗	769.9	-255.2, 1795.2	.14
Weight (kg)	97.4	74.1, 120.8	<.001∗	86.8	66.7, 106.9	<.001∗
BMI	274.2	182.3, 366.1	<.001∗			
Creatinine	-1570.1	-3766.1, 626.4	.16			
eGFR	65.1	31.5, 98.7	<.001∗	26.8	-0.63, 54.3	.06
AST	-48.7	-126.7, 29.3	.22			
ALT	10.8	-40.7, 62.4	.68			
Tbili	-2423.2	-5195.9, 349.6	.09			
Platelets	1.9	-9.4, 13.1	.74			
Baseline ACT	-79.9	-106.9, -52.8	<.001∗	-74.0	6902.2, 19,931.9	<.001∗

β = beta coefficient; AC = anticoagulant; ACT = activated clotting time; ALT = alanine transaminase; AST = aspartate aminotransferase; BMI = body mass index; eGFR = estimated glomerular filtration rate; Tbili = total bilirubin.

Example interpretation: a 1-unit increase in eGFR denotes a heparin bolus increase of 65.1 units.

Asterisk (∗) indicates statistically significant at *P* ≤ .05.

**Table 3:** Predictors of final heparin infusion rate

Variable	Univariate			Multivariate		
	β	95% CI	*P* value	β	95% CI	*P* value
Anticoagulation						
Apixaban	Ref	Ref	Ref	Ref	Ref	Ref
Warfarin	-883.2	-1525.6, -240.7	.007∗	-875.8	-1326.8, -424.8	<.001∗
Rivaroxaban	107.7	-553.1, 768.4	.75	-142.3	-615.6, 330.9	.55
Dabigatran	-1070.7	-2780.2, 638.9	.22	-476.1	-1678.6, 726.4	.43
INR (if on warfarin)	-514.5	-2245.4, 1216.4	.53			
Time since last AC dose to start	-0.2	-1.2, 0.9	.72			
Age	-31.8	-52.4, -11.2	.003∗	-7.28	-25.0, 10.5	.42
Sex = male	1170.3	787.8, 1552.9	<.001∗	580.3	253.6, 906.9	.001∗
Weight (kg)	33.0	26.8, 39.3	<.001∗	27.6	21.2, 33.9	<.001∗
BMI	81.2	54.4, 108.1	<.001∗			
Creatinine	-77.2	-724.9, 570.5	.81			
eGFR	13.6	3.5, 23.7	.009∗	3.77	-4.9, 12.4	.39
AST	-9.1	-31.8, 13.6	.43			
ALT	3.12	-11.9, 18.2	.68			
Tbili	-569.9	-1366.0, 226.1	.16			
Platelets	0.8	-2.5, 4.1	.64			
Baseline ACT	-7.8	-16.6, 0.91	.08			

β = beta coefficient; AC = anticoagulant; ACT = activated clotting time; ALT = alanine transaminase; AST = aspartate aminotransferase; BMI = body mass index; eGFR = estimated glomerular filtration rate; Tbili = total bilirubin.

Example interpretation: a 1-unit increase in eGFR denotes a heparin infusion increase of 13.6 units.

Asterisk (∗) indicates statistically significant at *P* ≤ .05.

### Complications

Complications included in the American Heart Association Get With the Guidelines AF Ablation registry are documented locally. There was a single reported complication in each arm: a pericardial effusion in the historical controls and a pseudoaneurysm requiring thrombin injection in the dosing algorithm arm.

## Discussion

The current work describes the development of a computerized, adaptive, rule-based heparin dosing algorithm to maintain therapeutic intraprocedural ACT during catheter ablation of AF. The dosing algorithm demonstrated benefits on the speed of reaching goal ACT and maintaining therapeutic anticoagulation compared to historical controls at the University of Wisconsin. The frequency at which the ACT dropped below the therapeutic range and rose into a supratherapeutic range was significantly reduced using our algorithm. Given the infrequent nature of clinically evident stroke / transient ischemic attack in recent reports (pooled incidence of 0.17%), it is difficult to demonstrate a hard outcome benefit with such a small study.^3^ However, Di Biase and colleagues^9^ did demonstrate silent cerebral injury was more frequent in a cohort of 428 patients when they did not have strict adherence to guideline-directed intraprocedural anticoagulation. This finding suggests that protocols that improve maintenance of therapeutic ACTs during AF ablation, like the one presented here, are important interventions to avoid significant procedural-related cerebral injury.

The present study also showed workflow improvements based on the usability score and subjective feedback provided by physicians and nurses. Staff and providers noted the benefits gained by removing the need for physician intervention for each heparin decision. These are very important findings. Gilmartin and colleagues^23^ demonstrated that Veterans Affairs cardiac catheterization laboratories that harnessed data to develop reliability enhancing work practices had higher staff job satisfaction, lower burnout, lower intent to leave, lower staff turnover, and higher perceived safety climate. Implementation of the present dosing algorithm represents one such attempt to create a reliability-enhancing practice that improved satisfaction in the University of Wisconsin electrophysiology laboratory.

Other centers have reported on the difficulty achieving therapeutic ACTs during catheter ablation for AF in patients on NOACs and have developed protocols to make improvements. Kishima and colleagues^15^ examined a retrospective cohort of 190 patients on NOACs and found that only 42% of patients reached an ACT >300 seconds at 30 minutes. After adjusting the protocol to include a higher initial bolus (100 U/kg + 5000 U) in patients with preablation ACT <130 seconds, this metric increased to 81%. In 89 patients on NOACs, Payne and colleagues^14^ found that 29% of ACTs were therapeutic when using an initial bolus of 120 U/kg and increased to 49% when the initial bolus was increased to 150 U/kg. Bradley and colleagues^16^ reported that a protocol-guided approach that accounted for preprocedure oral anticoagulant and weight-based dosing did increase the proportion of therapeutic ACT on first draw to 76.6%, from 57.7%, and decreased the average time to therapeutic ACT to 30 minutes; however, supratherapeutic ACT on first draw (>400 seconds) increased from 6.4% to 18.2%. Safani and colleagues^17^ reported impressive results using a comprehensive weight-adjusted, weight-based protocol that demonstrated a time to ACT >300 seconds of 14.6 minutes in 99 patients on NOACs. The authors used an adjusted weight with an initial bolus of 200 units/kg and infusion rate of 35 units/kg. In this study, all patients were on NOACs but over 75% of the patients were receiving dabigatran. While 90.8% of readings were ≥300 seconds, over 25% of all ACTs were more than 400 seconds.

The current study is notable for several reasons. The algorithm described here achieved time to goal ACT comparable to that shown by Safani and colleagues (17.6 min compared to 14.6 min) while reducing the frequency of ACT >400 seconds. The modeling suggests a path forward to improve initial heparin bolus and infusion amounts, possibly by including sex and eGFR in addition to baseline ACT, weight, and anticoagulant type. A larger retrospective model derivation and validation study is required prior to making these additions. Although the assessments of usability were unblinded and subjective, the user satisfaction reported is unique to this study and important to consider, given the findings from Gilmartin and colleagues.

Finally, the use of computerized CDSS accessible through the EMR is novel. As described by Sutton and colleagues,^24^ a computerized CDSS consists of the following: (1) a base—here, a set of rules and a mathematical function; (2) an interface engine, in this case a web-based interface opened through the EMR to input necessary clinical data; and (3) a communication mechanism: recommendations are communicated through the web-based interface. In the age of the EMR, attempts to develop effective CDSS are important to support the delivery of quality care. To date, many CDSS have targeted order sets, documentation templates, and patient-specific alerts.^25^ However, a task like heparin dosing in the electrophysiology lab lends itself to a computer-based CDSS, as most published guidance and protocols require repeated measurements and calculations that take into account specific patient characteristics like weight and anticoagulant type.^2^^,^^15^, ^16^, ^17^ The work here demonstrates a beginning, but there are limitations to be addressed and future work to be done to refine the tool.

### Limitations

This is a small, observational quality improvement study that describes the first implementation of this heparin dosing algorithm. The sample size is similar to other published reports but does limit the results.^14^, ^15^, ^16^, ^17^ The number of patients on each of the 4 preprocedural oral anticoagulants studied here is small—especially dabigatran—making the results less generalizable. When the ACT did drop below goal, the time below goal did decrease using the algorithm, but it was not a statistically significant decrease. The study was likely underpowered to establish this finding. The dosing algorithm requires initialization with a bolus amount and infusion rate. A larger sample size will allow for more refined derivation and validation of models to predict these values.

The lack of external validation limits generalizability and reproducibility. To mitigate potential bias, retrospective data collection (M.M.) and statistical analysis (F.O.) were performed by personnel outside of the electrophysiology team using the algorithm or the iVMD team that developed the dosing algorithm. However, those personnel were not blinded to the nature of the cases when doing analysis (control vs algorithm). To address these concerns, a multicenter randomized controlled trial with blinded analysis should be performed to confirm the findings of this study.

A future study should include objective measures of workflow improvement. The workflow improvements are key findings and require substantiation. Potential measures to include are time required for a decision regarding dosing, frequency of intraprocedural provider distractions, and the number of intraprocedural ACTs. Finally, it is critical to point out that this algorithm does require human oversight. The “fundamental theorem” of biomedical informatics states that persons supported by information technology will be better than the same person who is unassisted—not replaced by the information technology.^26^ That is especially true in this case, as errors in ACT measurement can be introduced owing to sample collection. Staff running ACT measurements using this algorithm will continue to review results and rerun samples if results are unexpected.

### Future directions

The current work is a starting point and represents 1 cycle in a continuous learning process (see Graphical Abstract).^27^ As has been described previously, there is individual variability in response to heparin that we do not fully understand.^12^ As shown in Tables 2 and 3, using the 139 dosing algorithm cases, we are beginning to understand factors that may contribute to this variability. A next step based on the modeling shown here will be to use a larger dataset to derive and test models for initial bolus and infusion rates that take into account sex and renal function in addition to anticoagulation type, weight, and baseline ACT.

Moving forward, it is also important to consider what the higher doses of heparin required for apixaban and rivaroxaban represent. A recent study by Martin and colleagues^28^ performed ex vivo testing using samples from patients on warfarin, patients on NOACs, or untreated patients. The authors argued that differences in ACT values observed for the different anticoagulants following a fixed dose of anticoagulant did not reflect a true difference in anticoagulant activity based on antithrombin concentrations (although those data were not shown) and that the higher doses of heparin may cause harm. This is an important discussion to continue within the electrophysiology community, and the result of that discussion could lead to a different target for intraprocedural anticoagulation.

No matter what the ultimate target is for anticoagulation, we feel that a heparin dosing algorithm delivered in a computer-based CDSS will have the potential to meet that target consistently while providing benefits to the workflow within the electrophysiology laboratory. After refining the initial heparin bolus and infusion amounts based on the modeling work described above, we look forward to a multi-institutional randomized controlled trial to address the limitations in the current report. A rigorous study done in a separate institution or multiple institutions that better defines how the dosing algorithm impacts workflows will be a critical step to better understand the effectiveness of the dosing algorithm.^24^^,^^25^

## Conclusion

The use of a deterministic, adaptive, rule-based dosing algorithm to dose intraprocedural heparin during AF ablation delivered as a computerized CDSS improved maintenance of therapeutic anticoagulation during AF ablation and provided subjective improvement in procedural workflows in this small, single-institution implementation.
