# Supplementary material for: Evaluation of an adaptive, rule-based dosing algorithm to maintain therapeutic anticoagulation during atrial fibrillation ablation
Source: Cardiovasc Digit Health J. 2023 Nov 14;4(6):173–82. doi: 10.1016/j.cvdhj.2023.11.001 (PMC10787148; doi:10.1016/j.cvdhj.2023.11.001)
Supplement: Supplemental Tables 1–6 [file mmc1.docx]

**SUPPLEMENTAL TABLES**

**Supplemental Table 1.** Initial Experience with Standard Bolus Amounts Scaled by Distance from Goal

| **Case** | **Weight** | **Drug** | **Initial ACT** | **Post Bolus ACT** |
| --- | --- | --- | --- | --- |
| Dev Part 1 - Case 1 | 104.3 | Apixaban | 116 | 275 |
| Dev Part 1 - Case 2 | 83.9 | Apixaban | 138 | 288 |
| Dev Part 1 - Case 3 | 84.4 | Apixaban | 152 | 379 |
| Dev Part 1 - Case 4 | 90.0 | Rivaroxaban | 158 | 313 |
| Dev Part 1 - Case 5 | 96.0 | Apixaban | 131 | 367 |
| Dev Part 1 - Case 6 | 81.6 | Warfarin | 151 | 379 |
| Dev Part 1 - Case 7 | 104.3 | Apixaban | 138 | 249 |
| Dev Part 1 - Case 8 | 132.0 | Apixaban | 110 | 243 |
| Dev Part 1 - Case 9 | 76.2 | Warfarin | 191 | 384 |
| Dev Part 1 - Case 10 | 83.9 | Apixaban | 138 | 246 |
| Dev Part 1 - Case 11 | 149.2 | Apixaban | 127 | 218 |
| Dev Part 1 - Case 12 | 72.6 | Rivaroxaban | 199 | 271 |
| Dev Part 1 - Case 13 | 78.0 | Apixaban | 162 | 368 |
| Dev Part 1 - Case 14 | 89.0 | Apixaban | 131 | 302 |
| Dev Part 1 - Case 15 | 93.0 | Apixaban | 145 | 368 |
| Dev Part 1 - Case 16 | 108.0 | Warfarin | 217 | 374 |
| Dev Part 1 - Case 17 | 61.0 | Apixaban | 157 | 271 |
| Dev Part 1 - Case 18 | 116.7 | Apixaban | 155 | 230 |

Rows highlighted in gray signify that post bolus ACT was < 300 seconds

**Supplemental Table 2.** Data Used to Refine Initial Bolus Amounts and Infusion Rates

| **Case** | **Weight** | **Drug** | **Initial ACT** | **Post Bolus ACT** | **Initial Infusion** | **Steady State Infusion** | **Steady State Infusion / Kg** | **Infusion Increase Factor** |
| --- | --- | --- | --- | --- | --- | --- | --- | --- |
| Dev Part 2 - Apix 1 | 93 | Apixaban | 161 | 246 | 2500 | 2500 | 26.9 | 1.00 |
| Dev Part 2 - Apix 2 | 90.3 | Apixaban | 128 | 331 | 1600 | 1600 | 17.7 | 1.00 |
| Dev Part 2 - Apix 3 | 63.5 | Apixaban | 136 | 417 | 900 | 900 | 14.2 | 1.00 |
| Dev Part 2 - Apix 4 | 113.4 | Apixaban | 129 | 295 | 2400 | 2550 | 22.5 | 1.06 |
| Dev Part 2 - Apix 5 | 86.7 | Apixaban | 121 | 389 | 1250 | 1600 | 18.5 | 1.28 |
| Dev Part 2 - Apix 6 | 70.8 | Apixaban | 131 | 279 | 1600 | 2150 | 30.4 | 1.34 |
| Dev Part 2 - Apix 7 | 140 | Apixaban | 138 | 269 | 3300 | 4450 | 31.8 | 1.35 |
| Dev Part 2 - Apix 8 | 65.3 | Apixaban | 134 | 286 | 1450 | 2000 | 30.6 | 1.38 |
| Dev Part 2 - Apix 9 | 100 | Apixaban | 121 | 284 | 2200 | 3200 | 32.0 | 1.45 |
| Dev Part 2 - Apix 10 | 140 | Apixaban | 138 | 269 | 3300 | 5000 | 35.7 | 1.52 |
| Dev Part 2 - Apix 11 | 93 | Apixaban | 130 | 344 | 1550 | 2350 | 25.3 | 1.52 |
| Dev Part 2 - Apix 12 | 75.8 | Apixaban | 131 | 318 | 1450 | 2200 | 29.0 | 1.52 |
| Dev Part 2 - Apix 13 | 127 | Apixaban | 130 | 249 | 3250 | 4950 | 39.0 | 1.52 |
| Dev Part 2 - Apix 14 | 67.1 | Apixaban | 143 | 372 | 1000 | 1550 | 23.1 | 1.55 |
| Dev Part 2 - Apix 15 | 78 | Apixaban | 136 | 268 | 1850 | 2950 | 37.8 | 1.59 |
| Dev Part 2 - Apix 16 | 128 | Apixaban | 144 | 333 | 2250 | 3700 | 28.9 | 1.64 |
| Dev Part 2 - Apix 17 | 104.3 | Apixaban | 121 | 276 | 2350 | 3900 | 37.4 | 1.66 |
| Dev Part 2 - Apix 18 | 79.4 | Apixaban | 133 | 368 | 1200 | 2050 | 25.8 | 1.71 |
| Dev Part 2 - Apix 19 | 69.8 | Apixaban | 137 | 366 | 1050 | 1800 | 25.8 | 1.71 |
| Dev Part 2 - Apix 20 | 88.5 | Apixaban | 136 | 321 | 1650 | 2850 | 32.2 | 1.73 |
| Dev Part 2 - Apix 21 | 104 | Apixaban | 141 | 357 | 1550 | 2700 | 26.0 | 1.74 |
| Dev Part 2 - Apix 22 | 95 | Apixaban | 141 | 318 | 1800 | 3200 | 33.7 | 1.78 |
| Dev Part 2 - Apix 23 | 81.6 | Apixaban | 151 | 376 | 1200 | 2150 | 26.3 | 1.79 |
| Dev Part 2 - Apix 24 | 118.4 | Apixaban | 125 | 374 | 1750 | 3150 | 26.6 | 1.80 |
| Dev Part 2 - Apix 25 | 104.3 | Apixaban | 144 | 344 | 1700 | 3200 | 30.7 | 1.88 |
| Dev Part 2 - Apix 26 | 79.4 | Apixaban | 125 | 294 | 1700 | 3350 | 42.2 | 1.97 |
| Dev Part 2 - Apix 27 | 81.6 | Apixaban | 115 | 335 | 1400 | 2800 | 34.3 | 2.00 |
| Dev Part 2 - Apix 28 | 149.7 | Apixaban | 132 | 347 | 2400 | 4950 | 33.1 | 2.06 |
| Dev Part 2 - Apix 29 | 114.3 | Apixaban | 128 | 258 | 1750 | 3750 | 32.8 | 2.14 |
| Dev Part 2 - Apix 30 | 104 | Apixaban | 142 | 358 | 1650 | 3750 | 36.1 | 2.27 |
| Dev Part 2 - Apix 31 | 106.6 | Apixaban | 128 | 351 | 1700 | 4150 | 38.9 | 2.44 |
| **Apixaban Summary** |  |  |  | **322** |  |  |  | **1.63** |
| Dev Part 2 - Riv 1 | 57.2 | Rivaroxaban | 198 | 320 | 1100 | 1550 | 27.1 | 1.41 |
| Dev Part 2 - Riv 2 | 88 | Rivaroxaban | 136 | 388 | 1300 | 2250 | 25.6 | 1.73 |
| Dev Part 2 - Riv 3 | 86.1 | Rivaroxaban | 180 | 310 | 1750 | 3350 | 38.9 | 1.91 |
| Dev Part 2 - Riv 4 | 114.3 | Rivaroxaban | 212 | 374 | 1650 | 1700 | 14.9 | 1.03 |
| Dev Part 2 - Riv 5 | 72 | Rivaroxaban | 191 | 316 | 1400 | 1950 | 27.1 | 1.39 |
| Dev Part 2 - Riv 6 | 84.4 | Rivaroxaban | 135 | 352 | 1350 | 2550 | 30.2 | 1.89 |
| Dev Part 2 - Riv 7 | 75 | Rivaroxaban | 168 | 288 | 1700 | 2150 | 28.7 | 1.26 |
| Dev Part 2 - Riv 8 | 109 | Rivaroxaban | 142 | 265 | 2800 | 4150 | 38.1 | 1.48 |
| Dev Part 2 - Riv 9 | 104.3 | Rivaroxaban | 127 | 259 | 2450 | 3200 | 30.7 | 1.31 |
| Dev Part 2 - Riv 10 | 83 | Rivaroxaban | 142 | 241 | 2200 | 2200 | 26.5 | 1.00 |
| Dev Part 2 - Riv 11 | 84.4 | Rivaroxaban | 91 | 224 | 1150 | 2700 | 32.0 | 2.35 |
| **Rivaroxaban Summary** |  |  |  | **303** |  |  |  | **1.52** |
| Dev Part 2 - Warf 1 | 89.7 | Warfarin | 114 | 284 | 1650 | 2350 | 26.2 | 1.42 |
| Dev Part 2 - Warf 2 | 97.5 | Warfarin | 170 | 382 | 1200 | 2000 | 20.5 | 1.67 |
| Dev Part 2 - Warf 3 | 88.5 | Warfarin | 164 | 334 | 1300 | 2050 | 23.2 | 1.58 |
| Dev Part 2 - Warf 4 | 84.8 | Warfarin | 171 | 354 | 1050 | 1850 | 21.8 | 1.76 |
| **Warfarin Summary** |  |  |  | **339** |  |  |  | **1.61** |
| Dev Part 2 - Dabi 1 | 97.5 | Dabigatran | 160 | 436 | 1450 | 1250 | 12.8 | 0.86 |
| Dev Part 2 - Dabi 2 | 80.7 | Dabigatran | 206 | 406 | 1200 | 1050 | 13.0 | 0.88 |
| **Dabigatran Summary** |  |  |  | **421** |  |  |  | **0.87** |

**Supplemental Table 3.** Intraprocedural anticoagulation outcomes using dosing algorithm compared to historical controls.

| **Outcome** | **Historical Control**  **N = 50** | **Dosing Algorithm**  **N = 139** | **Estimate (Algorithm), with HC as reference** | ***P* Value** |  |
| --- | --- | --- | --- | --- | --- |
| Time to goal ACT (>300S), Mean (SD) | 33.3 (23.6) | 17.6 (11.1) | β: -15.8 (-20.8, -10.8) | <0.001* |  |
| Average time <300s if ACT drifts below goal,  Mean (SD) | 25.5 (17.5) | 19.6 (10.0) | β: -5.9 (-13.7, 1.9) | 0.14 |  |
| Number of patients with any ACT >400s, n (%) | 10 (20.0) | 7 (5.0) | OR: 0.21 (0.07 – 0.59) | 0.003* |  |
| Number of patients with any ACT <300s while in LA,  n (%) | 27 (54.0) | 26 (18.7) | OR: 0.20 (0.10 – 0.39) | <0.001* |  |
| *Statistically significant at p≤0.05  SD = Standard deviation, β = Beta coefficient, OR = Odds ratio | | | | | |

**Supplemental Table 4.** Sample output from dosing algorithm (from case 99).

| Bolus Recommendation | Actual Bolus | Infusion Recommendation | Actual Infusion | ACT | Patient Weight | Anticoagulant |
| --- | --- | --- | --- | --- | --- | --- |
| 12000 | 12000 |  |  | 125 | 86.2 | Apixaban |
| 0 |  | 2000 | 2000 | 357 | 86.2 | Apixaban |
| 0 |  | 2300 | 2300 | 331 | 86.2 | Apixaban |
| 0 |  | 2250 | 2250 | 354 | 86.2 | Apixaban |
| 1000 | 1000 | 3000 | 3000 | 309 | 86.2 | Apixaban |
| 0 |  | 3100 | 3100 | 332 | 86.2 | Apixaban |
| 0 |  | 3000 | 3000 | 359 | 86.2 | Apixaban |
| 0 |  | 3200 | 3200 | 343 | 86.2 | Apixaban |
| 0 |  | 3350 | 3350 | 336 | 86.2 | Apixaban |
| 1000 | 0 | 3900 | 3900 | 311 | 86.2 | Apixaban |

**Supplemental Table 5.** All Activated Clotting Times for intraprocedural heparin dosing in historical controls.

| Case | Baseline ACT | ACT 1 | ACT 2 | ACT 3 | ACT 4 | ACT 5 | ACT 6 | ACT 7 | ACT 8 | ACT 9 | ACT 10 | ACT 11 | ACT 12 | ACT 13 | ACT 14 | ACT 15 | ACT 16 | ACT 17 | ACT 18 | ACT 19 | ACT 20 | ACT 21 | ACT 22 | ACT 23 | Number of ACT | Average ACT | Standard Deviation | Standard Dev Last 5 |
| --- | --- | --- | --- | --- | --- | --- | --- | --- | --- | --- | --- | --- | --- | --- | --- | --- | --- | --- | --- | --- | --- | --- | --- | --- | --- | --- | --- | --- |
| 1 | NR | 135 | 252 | **261** | **397** | **400** | **390** | **387** | **363** |  |  |  |  |  |  |  |  |  |  |  |  |  |  |  | 8 | 323.1 | 97.0 | 14.6 |
| 2 | NR | 219 | 201 | **280** | **363** | **329** | **380** | **466** | **366** | **370** |  |  |  |  |  |  |  |  |  |  |  |  |  |  | 9 | 330.4 | 84.0 | 50.7 |
| 3 | NR | 349 | **373** | **352** | **329** | **305** | **400** | **369** | **361** | **373** | **366** | **365** |  |  |  |  |  |  |  |  |  |  |  |  | 11 | 358.4 | 24.9 | 4.5 |
| 4 | NR | 274 | **262** | **355** | **271** | **382** | **374** |  |  |  |  |  |  |  |  |  |  |  |  |  |  |  |  |  | 6 | 319.7 | 56.3 | 57.8 |
| 5 | NR | 397 | **377** | **349** | **360** | **328** | **379** | **441** | **372** | **366** | **362** |  |  |  |  |  |  |  |  |  |  |  |  |  | 10 | 373.1 | 30.2 | 32.5 |
| 6 | NR | 411 | 397 | **349** | **369** | **366** | **360** | **354** | **322** | **355** |  |  |  |  |  |  |  |  |  |  |  |  |  |  | 9 | 364.8 | 26.2 | 17.1 |
| 7 | NR | 191 | **255** | **255** | **294** | **361** | **283** | **327** | **297** |  |  |  |  |  |  |  |  |  |  |  |  |  |  |  | 8 | 282.9 | 51.2 | 31.7 |
| 8 | NR | 194 | 373 | 394 | 291 | 360 | 368 | **351** | **348** | **355** | **366** | **420** | **305** | **273** |  |  |  |  |  |  |  |  |  |  | 13 | 338.3 | 59.2 | 56.9 |
| 9 | NR | 388 | 372 | **360** | **360** | **351** | **258** | **280** | **369** |  |  |  |  |  |  |  |  |  |  |  |  |  |  |  | 8 | 342.3 | 46.9 | 50.8 |
| 10 | NR | 371 | 362 | 318 | 363 | **349** | **343** | **369** | **366** | **382** | **382** | **379** | **370** |  |  |  |  |  |  |  |  |  |  |  | 12 | 362.8 | 18.5 | 7.4 |
| 11 | NR | 167 | 255 | 270 | 292 | **307** | **326** | **324** | **331** | **362** | **360** | **362** | **349** |  |  |  |  |  |  |  |  |  |  |  | 12 | 308.8 | 57.0 | 13.3 |
| 12 | NR | 368 | **293** | **305** | **295** | **355** | **309** | **366** | **368** | **371** | **357** | **363** |  |  |  |  |  |  |  |  |  |  |  |  | 11 | 340.9 | 32.6 | 5.3 |
| 13 | NR | 210 | 302 | **354** | **357** | **342** | **369** | **342** | **369** | **351** | **380** | **351** |  |  |  |  |  |  |  |  |  |  |  |  | 11 | 338.8 | 47.3 | 15.5 |
| 14 | NR | 401 | **376** | **360** | **358** | **371** | **360** | **357** | **349** | **348** | **363** | **354** | **419** | **357** |  |  |  |  |  |  |  |  |  |  | 13 | 367.2 | 20.8 | 28.9 |
| 15 | NR | 349 | 364 | **360** | **352** | **358** | **363** | **359** | **357** | **382** | **365** | **363** | **374** |  |  |  |  |  |  |  |  |  |  |  | 12 | 362.2 | 9.0 | 9.8 |
| 16 | NR | 358 | 351 | **302** | **352** | **368** | **360** | **373** | **374** | **355** | **354** |  |  |  |  |  |  |  |  |  |  |  |  |  | 10 | 354.7 | 20.3 | 9.7 |
| 17 | NR | 272 | **360** | **354** | **289** |  |  |  |  |  |  |  |  |  |  |  |  |  |  |  |  |  |  |  | 4 | 318.8 | 44.8 | 39.4 |
| 18 | NR | 418 | **390** | **371** | **368** | **377** | **360** | **383** | **371** |  |  |  |  |  |  |  |  |  |  |  |  |  |  |  | 8 | 379.8 | 18.0 | 8.8 |
| 19 | NR | 281 | **259** | **360** | **348** | **362** | **320** | **382** | **372** | **382** | **358** |  |  |  |  |  |  |  |  |  |  |  |  |  | 10 | 342.4 | 42.4 | 25.9 |
| 20 | NR | 231 | **299** | **323** | **330** | **317** | **366** | **380** | **361** | **360** | **419** | **385** | **373** |  |  |  |  |  |  |  |  |  |  |  | 12 | 345.3 | 49.2 | 24.3 |
| 21 | NR | 236 | **297** | **284** | **366** | **359** | **357** | **325** |  |  |  |  |  |  |  |  |  |  |  |  |  |  |  |  | 7 | 317.7 | 48.1 | 34.2 |
| 22 | NR | **328** | **298** | **357** | **363** | **368** | **379** | **407** | **388** |  |  |  |  |  |  |  |  |  |  |  |  |  |  |  | 8 | 361.0 | 34.4 | 17.5 |
| 23 | NR | 285 | 235 | **289** | **357** | **314** | **361** | **336** | **369** |  |  |  |  |  |  |  |  |  |  |  |  |  |  |  | 8 | 318.3 | 46.5 | 22.3 |
| 24 | NR | 352 | **318** | **355** | **351** | **334** | **325** | **376** | **369** | **366** | **370** |  |  |  |  |  |  |  |  |  |  |  |  |  | 10 | 351.6 | 20.0 | 20.6 |
| 25 | NR | 238 | **263** | **279** | **334** | **352** | **360** | **286** | **303** | **355** | **329** | **337** | **331** | **355** | **352** | **363** | **331** | **360** | **308** | **360** | **367** | **364** | **366** | **344** | 23 | 332.0 | 36.1 | 9.4 |
| 26 | NR | 349 | 357 | 295 | **368** | **360** | **348** | **330** | **344** | **368** | **372** | **358** |  |  |  |  |  |  |  |  |  |  |  |  | 11 | 349.9 | 21.9 | 17.4 |
| 27 | NR | 181 | **202** | **263** | **270** | **357** | **345** | **293** | **361** |  |  |  |  |  |  |  |  |  |  |  |  |  |  |  | 8 | 284.0 | 68.7 | 41.1 |
| 28 | NR | 199 | **182** | **248** | **348** | **324** | **348** | **278** | **332** |  |  |  |  |  |  |  |  |  |  |  |  |  |  |  | 8 | 282.4 | 66.6 | 28.8 |
| 29 | NR | 263 | **235** | **348** | **314** | **349** | **356** |  |  |  |  |  |  |  |  |  |  |  |  |  |  |  |  |  | 6 | 310.8 | 50.8 | 50.5 |
| 30 | NR | 204 | **241** | **323** | **355** | **329** | **364** | **360** | **331** | **371** | **354** | **366** |  |  |  |  |  |  |  |  |  |  |  |  | 11 | 327.1 | 54.8 | 16.6 |
| 31 | NR | 343 | 299 | **371** | **306** | **325** | **377** | **371** | **362** | **355** | **350** | **371** | **362** | **320** | **379** |  |  |  |  |  |  |  |  |  | 14 | 349.4 | 26.8 | 23.0 |
| 32 | NR | 199 | **219** | **287** | **352** | **355** | **317** | **311** |  |  |  |  |  |  |  |  |  |  |  |  |  |  |  |  | 7 | 291.4 | 61.3 | 28.9 |
| 33 | NR | 213 | **274** | **279** | **298** | **373** | **355** |  |  |  |  |  |  |  |  |  |  |  |  |  |  |  |  |  | 6 | 298.7 | 58.4 | 45.4 |
| 34 | NR | 277 | 238 | 372 | **352** | **357** | **296** | **388** | **358** |  |  |  |  |  |  |  |  |  |  |  |  |  |  |  | 8 | 329.8 | 52.9 | 33.5 |
| 35 | NR | 243 | **311** | **238** | **347** | **331** | **362** | **357** | **363** |  |  |  |  |  |  |  |  |  |  |  |  |  |  |  | 8 | 319.0 | 51.5 | 13.3 |
| 36 | NR | 241 | 279 | **295** | **368** | **266** | **260** | **334** | **360** | **387** | **357** |  |  |  |  |  |  |  |  |  |  |  |  |  | 10 | 314.7 | 52.4 | 48.3 |
| 37 | NR | 190 | 292 | 349 | **291** | **369** | **352** | **357** | **308** | **320** | **374** | **354** | **347** | **303** | **365** | **349** |  |  |  |  |  |  |  |  | 15 | 328.0 | 47.3 | 23.7 |
| 38 | NR | 319 | **355** | **346** | **344** | **328** | **366** | **377** | **366** | **346** | **364** | **360** | **356** | **322** |  |  |  |  |  |  |  |  |  |  | 13 | 349.9 | 18.0 | 16.8 |
| 39 | NR | 353 | 311 | **360** | **352** | **282** | **361** |  |  |  |  |  |  |  |  |  |  |  |  |  |  |  |  |  | 6 | 336.5 | 32.5 | 35.2 |
| 40 | NR | 362 | 333 | 329 | 355 | **352** | **330** | **358** | **358** | **357** | **354** |  |  |  |  |  |  |  |  |  |  |  |  |  | 10 | 348.8 | 12.8 | 12.1 |
| 41 | NR | 256 | **354** | **357** | **369** | **352** | **354** | **327** | **390** | **384** | **380** | **352** | **380** | **393** | **404** | **395** | **393** | **395** |  |  |  |  |  |  | 17 | 366.8 | 35.6 | 23.2 |
| 42 | NR | 283 | **351** | **352** | **334** | **354** | **380** | **366** | **365** | **366** |  |  |  |  |  |  |  |  |  |  |  |  |  |  | 9 | 350.1 | 28.3 | 9.2 |
| 43 | NR | 220 | **357** | **354** | **323** | **355** | **323** | **348** | **237** |  |  |  |  |  |  |  |  |  |  |  |  |  |  |  | 8 | 314.6 | 55.0 | 47.1 |
| 44 | NR | 182 | **306** | **293** | **365** | **262** | **298** | **351** | **352** | **357** | **367** | **368** | **363** |  |  |  |  |  |  |  |  |  |  |  | 12 | 322.0 | 56.8 | 6.8 |
| 45 | NR | 308 | 349 | **271** | **456** | **358** | **372** | **349** | **357** | **358** |  |  |  |  |  |  |  |  |  |  |  |  |  |  | 9 | 353.1 | 49.8 | 8.3 |
| 46 | NR | 348 | 292 | **349** | **296** | **363** | **346** | **357** | **357** | **354** | **332** |  |  |  |  |  |  |  |  |  |  |  |  |  | 10 | 339.4 | 25.4 | 10.6 |
| 47 | NR | 351 | **352** | **290** | **363** | **294** | **360** | **382** | **362** |  |  |  |  |  |  |  |  |  |  |  |  |  |  |  | 8 | 344.3 | 33.6 | 33.7 |
| 48 | NR | **315** | **349** | **345** | **321** | **352** | **344** | **313** | **361** | **355** | **323** |  |  |  |  |  |  |  |  |  |  |  |  |  | 10 | 337.8 | 17.9 | 20.6 |
| 49 | NR | 243 | 260 | **292** | **315** | **322** | **320** | **265** |  |  |  |  |  |  |  |  |  |  |  |  |  |  |  |  | 7 | 288.1 | 32.3 | 24.3 |
| 50 | NR | 349 | **318** | **360** | **361** | **327** | **352** | **355** | **363** | **357** | **357** | **358** |  |  |  |  |  |  |  |  |  |  |  |  | 11 | 350.6 | 14.6 | 3.0 |
| ACTs in BOLD represent ACTs in the LA | | | | | | | | | | | | | | | | | | | | | | Average | | | 9.9 | 334.4 | 40.9 | 24.6 |

**Supplemental Table 6.** All Activated Clotting Times for intraprocedural heparin dosing with dosing algorithm.

| Case | Baseline ACT | ACT 1 | ACT 2 | ACT 3 | ACT 4 | ACT 5 | ACT 6 | ACT 7 | ACT 8 | ACT 9 | ACT 10 | ACT 11 | ACT 12 | ACT 13 | ACT 14 | ACT 15 | ACT 16 | ACT 17 | ACT 18 | ACT 19 | Number of ACTs | Average ACT | Standard Deviation | Standard Dev Last 5 |
| --- | --- | --- | --- | --- | --- | --- | --- | --- | --- | --- | --- | --- | --- | --- | --- | --- | --- | --- | --- | --- | --- | --- | --- | --- |
| 1 | 111 | 232 | **268** | **312** | **309** | **323** | **347** | **345** | **327** | **324** | **336** | **331** | **330** |  |  |  |  |  |  |  | 12 | 315.3 | 33.4 | 4.5 |
| 2 | 135 | 318 | **352** | **303** | **348** | **374** | **366** | **369** | **365** | **357** |  |  |  |  |  |  |  |  |  |  | 9 | 350.2 | 24.3 | 6.2 |
| 3 | 135 | 307 | 314 | **355** | **352** | **310** | **352** | **360** | **355** | **360** | **349** |  |  |  |  |  |  |  |  |  | 10 | 341.4 | 21.8 | 4.9 |
| 4 | 129 | 366 | 366 | **331** | **318** | **343** | **344** | **346** | **324** | **357** | **367** | **355** | **322** | **355** | **360** | **357** |  |  |  |  | 15 | 347.4 | 16.7 | 15.7 |
| 5 | 174 | 330 | 276 | 333 | **310** | **354** | **354** | **352** | **344** | **357** | **361** |  |  |  |  |  |  |  |  |  | 10 | 337.1 | 26.5 | 6.3 |
| 6 | 179 | 376 | 379 | 366 | 380 | 372 | **358** | **365** | **352** | **354** | **374** | **366** |  |  |  |  |  |  |  |  | 11 | 367.5 | 9.7 | 9.1 |
| 7 | 132 | 243 | 357 | 364 | 357 | **309** | **352** | **352** | **324** | **352** | **342** | **316** | **346** | **378** | **357** | **352** | **327** | **304** | **353** |  | 18 | 338.1 | 30.9 | 22.7 |
| 8 | 101 | 294 | 357 | 349 | **310** | **323** | **358** | **332** | **347** | **294** |  |  |  |  |  |  |  |  |  |  | 9 | 329.3 | 25.5 | 24.6 |
| 9 | 118 | **248** | **223** | **354** | **321** | **331** | **322** | **335** | **352** |  |  |  |  |  |  |  |  |  |  |  | 8 | 310.8 | 48.5 | 12.6 |
| 10 | 182 | 393 | **371** | **375** | **355** | **357** | **325** | **323** |  |  |  |  |  |  |  |  |  |  |  |  | 7 | 357.0 | 25.8 | 22.4 |
| 11 | 121 | 221 | 281 | 331 | **352** | **357** | **360** | **360** | **336** | **352** | **357** | **357** | **348** | **354** | **327** | **362** |  |  |  |  | 15 | 337.0 | 38.2 | 13.6 |
| 12 | 136 | 352 | **328** | **352** | **326** | **333** | **347** | **344** | **308** |  |  |  |  |  |  |  |  |  |  |  | 8 | 336.3 | 15.4 | 15.7 |
| 13 | 170 | 327 | 320 | 355 | **352** | **361** | **347** | **332** | **331** | **326** | **345** | **344** |  |  |  |  |  |  |  |  | 11 | 340.0 | 13.5 | 8.4 |
| 14 | 151 | 290 | **316** | **334** | **318** | **355** | **348** | **315** |  |  |  |  |  |  |  |  |  |  |  |  | 7 | 325.1 | 22.2 | 17.7 |
| 15 | 164 | 312 | **343** | **309** | **349** | **343** | **355** |  |  |  |  |  |  |  |  |  |  |  |  |  | 6 | 335.2 | 19.6 | 17.9 |
| 16 | 164 | 317 | 356 | **335** | **352** | **323** | **366** | **380** | **368** |  |  |  |  |  |  |  |  |  |  |  | 8 | 349.6 | 22.6 | 21.8 |
| 17 | 140 | 385 | **306** | **283** | **351** | **352** | **361** | **351** | **363** | **333** | **333** | **357** |  |  |  |  |  |  |  |  | 11 | 343.2 | 28.4 | 13.8 |
| 18 | 157 | 311 | **294** | **343** | **328** | **346** | **363** |  |  |  |  |  |  |  |  |  |  |  |  |  | 6 | 330.8 | 25.2 | 26.0 |
| 19 | 125 | 346 | 261 | 261 | **351** | **351** | **350** | **345** | **326** | **351** | **344** | **323** |  |  |  |  |  |  |  |  | 11 | 328.1 | 34.6 | 12.5 |
| 20 | 156 | 275 | **349** | **349** | **326** | **351** | **355** | **355** | **349** |  |  |  |  |  |  |  |  |  |  |  | 8 | 338.6 | 27.3 | 12.1 |
| 21 | 135 | 246 | 330 | **314** | **375** | **343** | **326** | **357** |  |  |  |  |  |  |  |  |  |  |  |  | 7 | 327.3 | 41.2 | 24.2 |
| 22 | 219 | 244 | 363 | 354 | **379** | **315** | **351** | **363** | **357** | **354** |  |  |  |  |  |  |  |  |  |  | 9 | 342.2 | 40.6 | 19.0 |
| 23 | 114 | 302 | 349 | 303 | 333 | **336** | **347** | **334** | **354** |  |  |  |  |  |  |  |  |  |  |  | 8 | 332.3 | 19.9 | 14.1 |
| 24 | 152 | 357 | **348** | **322** | **334** | **336** | **331** | **302** | **323** |  |  |  |  |  |  |  |  |  |  |  | 8 | 331.6 | 16.8 | 13.9 |
| 25 | 136 | **301** | 332 | 328 | 301 | 366 | 369 |  |  |  |  |  |  |  |  |  |  |  |  |  | 6 | 332.8 | 29.9 | 28.5 |
| 26 | 131 | 344 | 331 | 294 | **327** | **352** | **302** | **354** | **380** | **362** | **369** | **371** | **370** | **365** |  |  |  |  |  |  | 13 | 347.8 | 27.1 | 3.8 |
| 27 | 180 | 348 | 349 | 352 | 352 | 295 | **357** | **332** | **345** | **346** | **322** | **325** |  |  |  |  |  |  |  |  | 11 | 338.5 | 18.4 | 11.1 |
| 28 | 162 | 349 | 318 | 348 | 324 | **352** | **334** | **349** | **352** | **352** | **360** | **358** |  |  |  |  |  |  |  |  | 11 | 345.1 | 13.7 | 4.6 |
| 29 | 147 | 307 | **344** | **323** | **313** | **343** | **349** | **358** | **344** | **355** |  |  |  |  |  |  |  |  |  |  | 9 | 337.3 | 18.4 | 6.6 |
| 30 | 127 | 277 | 357 | 349 | **318** | **363** | **357** | **355** | **352** | **349** | **366** |  |  |  |  |  |  |  |  |  | 10 | 344.3 | 27.0 | 6.5 |
| 31 | 138 | 248 | **348** | **333** | **308** | **357** | **322** | **352** | **365** | **330** |  |  |  |  |  |  |  |  |  |  | 9 | 329.2 | 35.4 | 18.3 |
| 32 | 142 | 284 | **326** | **296** | **355** | **366** | **314** | **364** | **367** | **368** | **352** | **291** |  |  |  |  |  |  |  |  | 11 | 334.8 | 33.4 | 32.7 |
| 33 | 138 | 299 | 308 | **304** | **349** | **354** | **305** | **357** | **358** |  |  |  |  |  |  |  |  |  |  |  | 8 | 329.3 | 27.2 | 22.4 |
| 34 | 138 | 363 | 345 | **348** | **308** | **346** | **355** | **343** |  |  |  |  |  |  |  |  |  |  |  |  | 7 | 344.0 | 17.3 | 18.4 |
| 35 | 171 | 353 | 259 | 379 | 349 | 346 | **374** | **368** | **377** | **382** | **368** |  |  |  |  |  |  |  |  |  | 10 | 355.5 | 36.2 | 6.0 |
| 36 | 142 | 357 | 307 | **358** | **318** | **355** | **363** | **349** | **331** | **358** |  |  |  |  |  |  |  |  |  |  | 9 | 344.0 | 20.3 | 12.4 |
| 37 | 135 | 363 | 327 | 310 | 349 | 354 | **342** | **347** | **320** | **330** | **375** | **358** |  |  |  |  |  |  |  |  | 11 | 343.2 | 19.7 | 21.9 |
| 38 | 168 | 286 | **352** | **354** | **321** | **348** | **333** | **341** |  |  |  |  |  |  |  |  |  |  |  |  | 7 | 333.6 | 24.0 | 12.9 |
| 39 | 132 | 277 | 362 | 331 | 320 | 374 | 360 | 362 | 368 | 368 | 376 | 398 | 381 | **393** | **393** | **431** | **390** | **394** |  |  | 17 | 369.3 | 35.0 | 17.3 |
| 40 | 174 | 309 | 285 | **375** | **263** | **375** | **381** | **367** |  |  |  |  |  |  |  |  |  |  |  |  | 7 | 336.4 | 49.5 | 50.1 |
| 41 | 174 | 305 | **280** | **330** | **304** | **332** | **266** | **391** | **406** | **403** | **376** |  |  |  |  |  |  |  |  |  | 10 | 339.3 | 51.6 | 58.4 |
| 42 | 118 | **313** | **272** | **274** | **335** | **351** | **329** |  |  |  |  |  |  |  |  |  |  |  |  |  | 6 | 312.3 | 32.8 | 36.7 |
| 43 | 141 | 233 | 371 | 384 | 365 | **366** | **366** | **363** | **360** | **366** |  |  |  |  |  |  |  |  |  |  | 9 | 352.7 | 45.4 | 2.7 |
| 44 | 159 | 351 | 305 | 317 | 357 | 328 | **344** | **283** | **366** | **358** | **349** | **364** | **355** | **363** | **369** |  |  |  |  |  | 14 | 343.5 | 25.8 | 7.9 |
| 45 | 143 | 320 | 255 | 334 | 303 | **344** | **318** | **349** | **329** | **358** | **324** | **352** |  |  |  |  |  |  |  |  | 11 | 326.0 | 28.8 | 15.0 |
| 46 | 144 | 285 | **317** | **345** | **357** | **354** | **352** | **360** |  |  |  |  |  |  |  |  |  |  |  |  | 7 | 338.6 | 27.7 | 5.7 |
| 47 | 156 | 350 | 328 | **312** | **366** | **361** | **375** | **355** | **352** | **329** |  |  |  |  |  |  |  |  |  |  | 9 | 347.6 | 20.5 | 16.7 |
| 48 | 152 | 271 | **312** | **352** | **325** | **349** | **308** | **349** | **342** |  |  |  |  |  |  |  |  |  |  |  | 8 | 326.0 | 28.1 | 17.8 |
| 49 | 187 | 322 | 379 | **377** | **359** | **374** | **357** | **371** | **362** |  |  |  |  |  |  |  |  |  |  |  | 8 | 362.6 | 18.4 | 7.5 |
| 50 | 152 | 332 | **291** | **349** | **333** | **321** | **311** | **372** | **354** | **351** | **357** | **360** | **354** |  |  |  |  |  |  |  | 12 | 340.4 | 23.4 | 3.4 |
| 51 | 147 | 357 | **347** | **334** | **346** | **333** | **343** | **324** | **355** |  |  |  |  |  |  |  |  |  |  |  | 8 | 342.4 | 11.4 | 12.0 |
| 52 | 138 | 322 | 336 | 312 | 326 | **317** | **346** | **350** | **352** | **357** | **352** |  |  |  |  |  |  |  |  |  | 10 | 337.0 | 16.6 | 4.0 |
| 53 | 135 | 365 | **305** | **317** | **354** | **358** | **358** | **361** |  |  |  |  |  |  |  |  |  |  |  |  | 7 | 345.4 | 24.0 | 18.4 |
| 54 | 138 | 361 | **301** | **366** | **333** | **311** | **328** | **349** | **357** | **357** | **333** | **323** |  |  |  |  |  |  |  |  | 11 | 338.1 | 21.5 | 15.2 |
| 55 | 197 | 257 | **352** | **366** | **323** | **405** | **401** | **413** | **404** |  |  |  |  |  |  |  |  |  |  |  | 8 | 365.1 | 53.9 | 37.3 |
| 56 | 139 | 363 | **349** | **310** | **303** | **362** | **352** | **330** | **349** | **352** | **351** |  |  |  |  |  |  |  |  |  | 10 | 342.1 | 20.8 | 9.5 |
| 57 | 130 | 293 | **319** | **360** | **371** | **352** | **357** |  |  |  |  |  |  |  |  |  |  |  |  |  | 6 | 342.0 | 29.7 | 19.6 |
| 58 | 225 | 358 | **330** | **350** | **328** | **306** | **336** | **364** | **358** |  |  |  |  |  |  |  |  |  |  |  | 8 | 341.3 | 19.7 | 23.5 |
| 59 | 157 | 332 | **299** | **333** | **322** | **352** | **355** | **361** | **361** | **346** | **357** | **349** |  |  |  |  |  |  |  |  | 11 | 342.5 | 19.3 | 6.9 |
| 60 | 131 | 307 | **314** | **348** | **332** | **351** | **306** | **357** |  |  |  |  |  |  |  |  |  |  |  |  | 7 | 330.7 | 21.8 | 20.5 |
| 61 | 164 | 327 | 304 | 240 | 244 | **337** | **371** | **330** | **360** | **372** | **369** | **360** |  |  |  |  |  |  |  |  | 11 | 328.5 | 47.9 | 16.6 |
| 62 | 138 | 332 | 303 | **368** | **357** | **357** |  |  |  |  |  |  |  |  |  |  |  |  |  |  | 5 | 343.4 | 26.2 | 26.2 |
| 63 | 153 | 314 | 332 | 300 | **306** | **344** | **347** | **363** | **349** |  |  |  |  |  |  |  |  |  |  |  | 8 | 331.9 | 22.8 | 21.3 |
| 64 | 178 | 355 | 241 | **366** | **363** | **323** | **372** | **373** | **321** | **403** | **381** |  |  |  |  |  |  |  |  |  | 10 | 349.8 | 45.6 | 30.1 |
| 65 | 159 | 305 | 347 | 304 | **303** | **359** | **347** | **366** | **345** | **350** |  |  |  |  |  |  |  |  |  |  | 9 | 336.2 | 25.1 | 8.8 |
| 66 | 134 | 259 | 334 | **349** | **323** | **334** | **297** |  |  |  |  |  |  |  |  |  |  |  |  |  | 6 | 316.0 | 32.9 | 19.3 |
| 67 | 155 | 345 | 355 | 314 | **351** | **351** | **329** | **336** | **336** | **350** | **314** | **361** |  |  |  |  |  |  |  |  | 11 | 340.2 | 15.9 | 17.7 |
| 68 | 133 | 380 | 355 | 357 | **349** | **346** | **313** | **333** | **343** | **361** |  |  |  |  |  |  |  |  |  |  | 9 | 348.6 | 18.7 | 17.8 |
| 69 | 158 | 352 | 347 | **315** | **334** | **331** | **324** | **361** | **349** | **361** | **349** | **335** | **349** | **351** |  |  |  |  |  |  | 13 | 342.9 | 14.0 | 9.3 |
| 70 | 149 | 399 | **362** | **304** | **355** | **354** | **348** | **330** |  |  |  |  |  |  |  |  |  |  |  |  | 7 | 350.3 | 29.2 | 21.6 |
| 71 | 132 | 327 | **311** | **344** | **354** | **325** | **343** | **324** | **355** | **343** |  |  |  |  |  |  |  |  |  |  | 9 | 336.2 | 15.1 | 13.3 |
| 72 | 182 | 360 | 354 | 355 | **302** | **305** | **369** | **321** | **369** |  |  |  |  |  |  |  |  |  |  |  | 8 | 341.9 | 28.0 | 33.9 |
| 73 | 122 | 312 | **334** | **307** | **301** | **355** | **354** | **361** | **309** | **372** | **331** | **325** |  |  |  |  |  |  |  |  | 11 | 332.8 | 24.5 | 26.1 |
| 74 | 142 | 359 | **363** | **349** | **353** | **344** | **347** | **306** | **358** |  |  |  |  |  |  |  |  |  |  |  | 8 | 347.4 | 17.9 | 20.6 |
| 75 | 138 | 354 | **357** | **346** | **301** | **361** | **352** |  |  |  |  |  |  |  |  |  |  |  |  |  | 6 | 345.2 | 22.2 | 24.4 |
| 76 | 162 | 319 | **307** | **322** | **360** | **349** | **355** | **328** | **349** |  |  |  |  |  |  |  |  |  |  |  | 8 | 336.1 | 19.5 | 12.2 |
| 77 | 151 | **374** | **363** | **333** | **348** | **343** | **344** | **348** | **346** | **346** | **326** | **316** |  |  |  |  |  |  |  |  | 11 | 344.3 | 15.9 | 14.5 |
| 78 | 139 | 360 | 297 | 303 | 357 | **356** | **364** | **349** | **333** | **321** | **347** | **347** |  |  |  |  |  |  |  |  | 11 | 339.5 | 23.1 | 12.1 |
| 79 | 119 | 303 | 317 | **312** | **355** | **349** | **332** | **354** | **345** |  |  |  |  |  |  |  |  |  |  |  | 8 | 333.4 | 20.4 | 9.3 |
| 80 | 210 | 225 | **355** | **334** | **308** | **357** | **355** | **331** | **349** | **352** | **332** | **335** |  |  |  |  |  |  |  |  | 11 | 330.3 | 37.9 | 9.9 |
| 81 | 136 | 241 | **329** | **387** | **368** | **358** | **330** | **321** | **358** | **270** |  |  |  |  |  |  |  |  |  |  | 9 | 329.1 | 47.2 | 36.1 |
| 82 | 133 | 301 | 255 | **336** | **346** | **306** | **331** | **294** | **326** | **290** |  |  |  |  |  |  |  |  |  |  | 9 | 309.4 | 28.4 | 18.5 |
| 83 | 138 | 352 | **311** | **312** | **318** | **355** | **353** | **306** | **354** | **346** | **354** |  |  |  |  |  |  |  |  |  | 10 | 336.1 | 21.3 | 20.7 |
| 84 | 127 | 390 | 374 | 365 | **363** | **372** | **357** | **354** | **372** | **360** |  |  |  |  |  |  |  |  |  |  | 9 | 367.4 | 11.0 | 8.5 |
| 85 | 129 | 273 | 313 | **354** | **330** | **349** | **307** |  |  |  |  |  |  |  |  |  |  |  |  |  | 6 | 321.0 | 30.1 | 20.9 |
| 86 | 143 | 301 | 293 | 369 | 354 | **343** | **345** | **335** | **355** | **316** |  |  |  |  |  |  |  |  |  |  | 9 | 334.6 | 25.9 | 14.6 |
| 87 | 152 | 349 | **323** | **317** | **283** | **343** | **372** | **358** | **363** |  |  |  |  |  |  |  |  |  |  |  | 8 | 338.5 | 29.3 | 35.6 |
| 88 | 138 | 245 | 292 | **302** | **353** | **337** | **330** | **334** | **349** |  |  |  |  |  |  |  |  |  |  |  | 8 | 317.8 | 36.3 | 9.9 |
| 89 | 132 | 354 | 355 | 358 | 328 | **335** | **317** | **357** | **352** | **355** | **369** | **346** | **325** |  |  |  |  |  |  |  | 12 | 345.9 | 15.9 | 16.0 |
| 90 | 198 | 354 | 326 | **347** | **327** | **364** | **351** |  |  |  |  |  |  |  |  |  |  |  |  |  | 6 | 344.8 | 15.3 | 16.3 |
| 91 | 136 | 316 | 268 | **308** | **333** | **352** | **349** | **355** |  |  |  |  |  |  |  |  |  |  |  |  | 7 | 325.9 | 31.3 | 19.5 |
| 92 | 128 | 347 | 331 | **305** | **315** | **375** | **367** | **363** | **310** |  |  |  |  |  |  |  |  |  |  |  | 8 | 339.1 | 27.7 | 30.9 |
| 93 | 135 | 349 | **320** | **352** | **316** | **354** | **354** | **362** | **352** |  |  |  |  |  |  |  |  |  |  |  | 8 | 344.9 | 17.0 | 18.1 |
| 94 | 137 | 364 | **332** | **363** | **345** | **352** | **313** | **301** | **363** | **366** | **354** |  |  |  |  |  |  |  |  |  | 10 | 345.3 | 22.9 | 30.2 |
| 95 | 165 | 357 | 354 | **358** | **355** | **336** | **321** |  |  |  |  |  |  |  |  |  |  |  |  |  | 6 | 346.8 | 15.0 | 15.9 |
| 96 | 144 | 347 | 314 | **347** | **318** | **351** | **355** | **352** | **357** |  |  |  |  |  |  |  |  |  |  |  | 8 | 342.6 | 16.8 | 16.2 |
| 97 | 126 | 153 | 404 | **389** | **394** | **366** | **377** | **399** | **365** |  |  |  |  |  |  |  |  |  |  |  | 8 | 355.9 | 83.3 | 15.7 |
| 98 | 136 | 318 | **332** | **327** | **352** |  |  |  |  |  |  |  |  |  |  |  |  |  |  |  | 4 | 332.3 | 14.4 | 14.4 |
| 99 | 125 | 357 | 331 | **354** | **309** | **332** | **359** | **343** | **336** | **311** |  |  |  |  |  |  |  |  |  |  | 9 | 336.9 | 18.5 | 17.5 |
| 100 | 190 | 273 | 290 | 372 | 366 | 366 | **381** | **369** | **369** | **371** | **343** |  |  |  |  |  |  |  |  |  | 10 | 350.0 | 37.6 | 14.1 |
| 101 | 143 | 386 | **377** | **355** | **366** | **352** | **351** | **349** | **333** |  |  |  |  |  |  |  |  |  |  |  | 8 | 358.6 | 16.9 | 11.7 |
| 102 | 153 | 352 | **345** | **350** | **300** | **335** | **360** | **346** | **358** | **324** |  |  |  |  |  |  |  |  |  |  | 9 | 341.1 | 19.0 | 15.3 |
| 103 | 152 | 361 | 355 | 357 | 352 | 355 | **326** | **343** | **355** | **329** | **361** | **327** | **327** |  |  |  |  |  |  |  | 12 | 345.7 | 14.4 | 16.8 |
| 104 | 153 | 281 | 349 | **311** | **347** | **355** | **358** | **346** | **357** | 284 |  |  |  |  |  |  |  |  |  |  | 9 | 332.0 | 31.4 | 31.7 |
| 105 | 145 | 352 | **286** | **322** | **321** | **330** | **293** | **358** | **354** | **321** | **360** | **358** | **357** | **314** | **337** |  |  |  |  |  | 14 | 333.1 | 24.7 | 19.8 |
| 106 | 138 | 354 | 321 | **295** | **335** | **308** | **324** | **350** | **349** | **357** |  |  |  |  |  |  |  |  |  |  | 9 | 332.6 | 22.0 | 20.7 |
| 107 | 130 | 231 | 244 | **224** | **287** | **331** | **312** | **331** | **330** | **325** |  |  |  |  |  |  |  |  |  |  | 9 | 290.6 | 45.6 | 8.1 |
| 108 | 190 | 280 | 269 | **345** | **284** | **325** | **369** | **372** |  |  |  |  |  |  |  |  |  |  |  |  | 7 | 320.6 | 43.3 | 36.2 |
| 109 | 132 | 345 | 318 | 357 | **335** | **352** | **344** | **360** | **328** | **351** |  |  |  |  |  |  |  |  |  |  | 9 | 343.3 | 13.9 | 12.0 |
| 110 | 125 | 355 | 330 | **348** | **332** | **271** | **321** | **368** | **378** | **355** |  |  |  |  |  |  |  |  |  |  | 9 | 339.8 | 31.7 | 43.5 |
| 111 | 159 | 304 | 288 | 352 | 343 | 333 | 248 | **250** | **380** | **355** |  |  |  |  |  |  |  |  |  |  | 9 | 317.0 | 47.2 | 60.9 |
| 112 | 143 | 293 | 285 | 343 | 302 | 310 | **360** | **352** | **355** | **355** | **361** |  |  |  |  |  |  |  |  |  | 10 | 331.6 | 30.4 | 3.8 |
| 113 | 150 | 285 | 349 | 351 | 351 | **321** | **352** | **349** | **349** | **328** | **358** | **357** | **357** | **355** |  |  |  |  |  |  | 13 | 343.2 | 20.7 | 12.9 |
| 114 | 149 | 314 | **358** | **321** | **329** | **323** |  |  |  |  |  |  |  |  |  |  |  |  |  |  | 5 | 329.0 | 17.1 | 17.1 |
| 115 | 152 | 322 | 285 | **358** | **349** | **319** | **352** | **352** | **355** | **359** | **355** | **366** | **349** | **352** | **360** |  |  |  |  |  | 14 | 345.2 | 21.9 | 6.7 |
| 116 | 181 | 265 | **368** | **400** | **371** | **381** |  |  |  |  |  |  |  |  |  |  |  |  |  |  | 5 | 357.0 | 52.9 | 52.9 |
| 117 | 152 | 205 | 196 | 245 | 291 | **305** | **310** | **307** | **317** | **306** | **365** | **311** | **354** | **309** | **331** | **349** | **302** | **348** | **352** | **347** | 19 | 307.9 | 47.3 | 21.1 |
| 118 | 252 | 273 | 386 | 381 | **374** | **389** | **380** | **377** | **361** |  |  |  |  |  |  |  |  |  |  |  | 8 | 365.1 | 38.2 | 10.2 |
| 119 | 169 | 366 | **361** | **351** | **363** | **357** | **351** | **357** |  |  |  |  |  |  |  |  |  |  |  |  | 7 | 358.0 | 5.7 | 5.0 |
| 120 | 163 | 376 | 358 | 357 | **363** | **363** | **357** |  |  |  |  |  |  |  |  |  |  |  |  |  | 6 | 362.3 | 7.3 | 3.1 |
| 121 | 150 | 374 | 351 | **360** | **334** | **334** | **368** | **304** | **354** | **351** | **352** | **363** | **354** |  |  |  |  |  |  |  | 12 | 349.9 | 18.7 | 4.8 |
| 122 | 127 | 382 | **366** | **369** | **379** | **391** | **416** | **388** |  |  |  |  |  |  |  |  |  |  |  |  | 7 | 384.4 | 16.7 | 17.6 |
| 123 | 133 | 258 | **374** | **404** | **351** | **349** | **333** |  |  |  |  |  |  |  |  |  |  |  |  |  | 6 | 344.8 | 49.2 | 27.6 |
| 124 | 174 | 349 | 357 | **348** | **333** | **328** | **330** | **327** | **349** | **321** |  |  |  |  |  |  |  |  |  |  | 9 | 338.0 | 12.8 | 10.6 |
| 125 | 163 | 329 | 267 | **300** | **347** | **318** | **349** | **352** | **344** | **355** | **349** |  |  |  |  |  |  |  |  |  | 10 | 331.0 | 28.5 | 4.1 |
| 126 | 135 | 302 | 343 | 280 | **357** | **349** | **351** | **364** | **380** | **372** | **377** |  |  |  |  |  |  |  |  |  | 10 | 347.5 | 32.6 | 11.6 |
| 127 | 126 | 372 | 358 | **363** | **357** | **354** | **363** |  |  |  |  |  |  |  |  |  |  |  |  |  | 6 | 361.2 | 6.4 | 3.9 |
| 128 | 152 | 349 | 299 | **314** | **304** | **335** | **329** | **349** | **352** | **343** | **344** | **352** |  |  |  |  |  |  |  |  | 11 | 333.6 | 19.6 | 4.3 |
| 129 | 146 | 286 | **323** | **319** | **355** | **352** | **357** | **332** | **257** |  |  |  |  |  |  |  |  |  |  |  | 8 | 322.6 | 35.5 | 42.3 |
| 130 | 179 | 311 | **254** | **368** | **357** | **361** | **363** | **362** | **360** |  |  |  |  |  |  |  |  |  |  |  | 8 | 342.0 | 39.9 | 2.3 |
| 131 | 179 | 332 | 319 | **352** | **363** | **349** | **317** | **366** | **374** | **361** | **373** | **392** | **366** | **372** |  |  |  |  |  |  | 13 | 356.6 | 22.3 | 11.8 |
| 132 | 135 | 278 | **352** | **358** | **355** | **357** | **356** | **352** |  |  |  |  |  |  |  |  |  |  |  |  | 7 | 344.0 | 29.2 | 2.3 |
| 133 | 124 | 287 | **314** | **378** | **307** | **297** | **328** | **333** | **317** | **364** | **369** | **363** | **387** |  |  |  |  |  |  |  | 12 | 337.0 | 33.9 | 25.9 |
| 134 | 140 | 304 | 354 | **358** | **354** | **349** | **347** | **353** | **298** | **355** | **354** |  |  |  |  |  |  |  |  |  | 10 | 342.6 | 22.2 | 24.5 |
| 135 | 153 | **301** | **349** | **317** | **307** | **360** | **354** | **349** | **332** | **294** | **368** |  |  |  |  |  |  |  |  |  | 10 | 333.1 | 26.6 | 28.5 |
| 136 | 157 | **316** | **287** | **347** | **352** | **329** | **320** | **355** | **332** | **371** |  |  |  |  |  |  |  |  |  |  | 9 | 334.3 | 25.1 | 21.0 |
| 137 | 140 | 358 | **357** | **354** | **332** | **352** | **343** |  |  |  |  |  |  |  |  |  |  |  |  |  | 6 | 349.3 | 10.0 | 10.2 |
| 138 | 144 | 377 | 366 | **357** | **361** | **371** | **358** | **349** | **349** |  |  |  |  |  |  |  |  |  |  |  | 8 | 361.0 | 10.0 | 9.2 |
| 139 | 139 | 379 | 370 | 363 | 372 | **352** | **352** | **349** | **354** | **349** |  |  |  |  |  |  |  |  |  |  | 9 | 360.0 | 11.3 | 2.2 |
| ACTs in **BOLD** represent ACTs in the LA | | | | | | | | | | | | | | | | | | Average | | | 9.2 | 339.8 | 26.3 | 17.3 |
